# Are menopause, aging and prostate cancer diseases?

**DOI:** 10.18632/aging.204499

**Published:** 2023-01-26

**Authors:** Mikhail V. Blagosklonny

**Affiliations:** 1 Roswell Park Comprehensive Cancer Center, Buffalo, NY 14263, USA

**Keywords:** geroscience, mTOR, hyperfunction theory of aging, lifespan, healthspan

## Abstract

There is no doubt that prostate cancer is a disease. Then, according to hyperfunction theory, menopause is also a disease. Like all age-related diseases, it is a natural process, but is also purely harmful, aimless and unintended by nature. But exactly because these diseases (menopause, prostate enlargement, obesity, atherosclerosis, hypertension, diabetes, presbyopia and thousands of others) are partially quasi-programmed, they can be delayed by slowing aging. Is aging a disease? Aging is a quasi-programmed disease that is partially treatable by rapamycin. On the other hand, aging is an abstraction, a sum of all quasi-programmed diseases and processes. In analogy, the zoo consists of animals and does not exist without animals, but the zoo is not an animal.

## Prostate cancer

Prostate cancer is an age-related disease. Every man would be diagnosed with prostate cancer, except that most men do not live long enough, dying from other age-related diseases. The frequency of prostate cancer detected by autopsy is 30-fold higher than mortality from prostate cancer so that “more men die with prostate cancer than because of it” [1]. Among men aged 70–79, a tumor is found by autopsy in 36% of Caucasians and 51% of African-Americans [1, 2]. The older the man, the higher frequency of autopsy-detected prostate cancer. The frequency of high-grade prostate cancer doubles every ten years [1].

Puberty is critical for susceptibility to prostate cancer later in life [3]. Older age at sexual maturation is linked to a decreased risk of prostate cancer later [4, 5]. Thus, prostate cancer is partially quasi-programmed (it will be discussed later) in puberty and would develop almost in everyone, if other causes of death did not exist.

## Prostate enlargement or BPH

Benign prostatic hyperplasia (BPH) is the most common age-related disease in men. An enlarged prostate can block the urethra, leading to an inability to urinate and kidney damage and, if left untreated, to death. Benign prostatic hyperplasia can be detectable by the age of 30. Between 30 and 50 the prostate grows in size, with a doubling time of 4.5 years. Between 51 and 70 years old, the doubling time is around 10 years [6]. Thus, the prostate is enlarged in every aging man, and therefore it is a “normal” disease, occurring in everyone, often asymptomatic.

Early in puberty, the prostate doubles in size, and its secretory function is increased to produce prostate fluid. During puberty, the prostate reaches the required size and function, but it continues to grow without purpose, becoming eventually hypertrophic, hyperplasic and hyper functional. The disease is quasi-programmed, a continuation of the developmental growth and reproductive program that was not switched off upon its completion. Quasi-programs are purely harmful and unintended by nature, but they are a continuation (or a byproduct) of essential programs, so natural selection is powerless to eliminate them. (Note: The force of natural selection is negligible late in life, so selection is very weak against quasi-programs. Natural selection is maximally strong for growth and reproductive programs, and quasi-programs are by-products).

Cellular hyperfunctions drive prostate growth and, ultimately, benign prostate hyperplasia (BPH). Hyperproliferation of epithelial and stromal cells, leukocyte infiltration, inflammation and other hyper-functions lead to BPH. Hypersecretory phenotype (hyperfunction) also known as senescence-associated secretory phenotype (SASP) contributes to the development of BPH [7, 8]. Prostatic inflammation (hyperfunction) stimulates prostatic growth and progression of symptoms [9].

mTOR drives cellular size growth, hyper-inflammation, senescent and hyper-secretory phenotypes [10–17]. Therefore, rapamycin (Rapatar) prevents prostate hypertrophy and hyperplasia and reduces inflammation in rat models of BPH [18].

## Atherosclerosis

Atherosclerosis is driven by hyperfunction of numerous cell types, acting locally and distantly. Thus, activation of endothelial cells, smooth muscle cells (SMC) and macrophages contributes to the formation of atherosclerotic plaque. Hypertrophy and hyperplasia of SMC and hypertrophic transformation of macrophages (foam cells) are hallmarks of atherosclerosis. Hyperfunctional blood platelets interact with the arterial wall, accelerating atherosclerosis and thrombosis. Adipocytes and hepatocytes hyperproduce atherogenic lipoproteins and cytokines. Hyperlipidemia, hyperglycemia, hyperinsulinemia, and hypertension contribute to atherosclerosis. Atherosclerosis is associated with all other diseases of aging, especially hypertension, type II diabetes and obesity.

Atherosclerosis originates in childhood and progresses throughout life [19]. It occurs in everyone. It is a hallmark of aging and a “normal disease”.

Clinical manifestations of atherosclerosis, cardiovascular diseases, are the main causes of death in humans. The path from cellular hyperfunction that causes atherosclerosis, hypertension and thrombosis to myocardial infarction is shown in Figure 2 in ref. [20].

Rapamycin (sirolimus) and its analog (everolimus) attenuate atherosclerosis in mice [21] and rabbits [22]. According to a prospective randomized controlled trial, rapamycin (sirolimus) decreased carotid atherosclerosis in humans [23].

## Menopause

Some age-related diseases are so program-like that they are considered to be the norm. Menopause happens in every woman (the average age at menopause is 51, according to the North American Menopause Society), and therefore it is not commonly viewed as a disease. But atherosclerosis and prostate enlargement (and all age-related diseases) also happen in everyone. One may argue that menopause is not as deadly as cancer. However, it is deadlier than osteoarthritis and Alzheimer’s disease. Menopause promotes cardiovascular diseases (CVD) osteoporosis, obesity, type II diabetes and other diseases [24, 25]. Needless to say, loss of reproductive function is highly disadvantageous from an evolutionary point of view (we will discuss the grandmother hypothesis in the next section).

One may argue that menopause occurs too early in life compared with prostate cancer and Alzheimer’s disease, for instance, to be called disease. However, premature menopause is considered a disease. By arbitrary definition, it occurs before the age of 40 years, or two standard deviations in years before the mean menopausal age of the study population [26].

Regulation of the menstrual cycle is very intricate and vulnerable, and hormonal hyperstimulation can disrupt the cycle. Even low doses of estradiol and progesterone are contraceptive. The famous contraceptive *“Plan B*”, a progestin, disrupts the menstrual cycle and prevents pregnancy by a single dose. (Note: in comparison, the regulation of a male reproduction function is much simpler, explaining why men do not lose it as much as women do with age).

Not surprisingly, hyperfunction of the hypothalamic-pituitary-ovarian axis eventually dysregulates the system and causes ovarian failure (see Figure 3 in ref. [27]). The menstrual cycle is tightly-regulated by numerous hormones, cell types and organs. Luteinizing hormone (LH) and follicle-stimulating hormone (FSH), produced by the pituitary gland, stimulate ovulation and the production of estrogens and progesterone by the ovary. For example, FSH stimulates follicles, production of ova and estrogens. Before puberty, the levels of both FSH and estrogens are low. To start the menstrual cycle, production of FSH is increased, stimulating the ovaries and estrogen production. Activation of follicles from the dormant pool serves as the source of fertilizable ova. With age, levels of FSH continuously increase, hyper-stimulating the ovaries [28], causing more follicles to be recruited simultaneously (see Figures 3–4 in ref. [27]).

Hyper-stimulation of follicle recruitment leads to follicular depletion and ovarian failure.

Thus, stimulation of FSH is initiates puberty, and its continuous hyperfunction accelerates menopause. Since the quasi-program of menopause is a continuation of puberty, mTOR, a central regulator of the onset of puberty, accelerates the onset of both puberty [29] and menopause in animals [30–32].

By activating mTOR, obesity accelerates ovarian follicle development and follicle loss in rats [33]. By inhibiting mTOR, calorie restriction delays puberty and extends reproductive lifespan in rodents [34, 35]. Overactivated mTOR activates the entire primordial follicle pool, and, subsequently, all primordial follicles become depleted in early adulthood, causing premature ovarian failure (POF) in mice [30–32].

Rapamycin preserves the follicle pool reserve and prolongs the ovarian lifespan in female rats [36] and mice [34, 37, 38]. mTOR is overactivated in the peripheral blood cells of women with premature ovarian insufficiency [39].

## Critique of the grandmother (great-great-grandmother hypothesis) hypothesis

As we discussed in the previous section, menopause is a byproduct of the reproductive program that initiates puberty. The same process that turns the menstrual cycle on in puberty becomes hyperfunctional, damaging the reproductive system and (unintentionally) switching it off. As are all age-related diseases, menopause is purely harmful and provides no benefits.

Some prominent gerontologists, however, hypothesize that menopause is adaptive and intended by natural selection to prevent older women from reproduction and thus redirect their efforts to help daughters to raise grandchildren [40]. Unless a daughter is a modern working mom, rather than a prehistorical female, this hypothesis makes no sense.

First, the natural age of grandmothers is 28, whereas menopause occurs at 51. Then the hypothesis should be renamed as great-great-grandmother hypothesis. The genetic similarities of a woman with great-grandchildren are less than with nephews and nieces.

Second, the best possible help would be breast feeding. However, post-menopausal women cannot get pregnant and therefore cannot lactate. If nature selects for caring for grandchildren, elderly women should produce milk or become pregnant to produce it.

Third, only maternal grandmothers increase grandchildren’s survival, whereas paternal mothers decrease it. The presence of paternal grandmothers (mothers-in-law) is detrimental to grandchild survival or well-being [41–44]. In most societies, a wife would likely live with a parental grandmother.

Fourth, only a minority of pre-historical females lived long enough to become great-great-grandmothers. Even 300 years ago in England, only 25% of people survived to the age of 26. How many would survive until menopause? It is commonly argued that hunter-gatherers lived as long as modern people. Although the maximal lifespan can be the same, due to accidental causes of death, the median lifespan of any species in the wild is much shorter than in a protected environment (laboratory animals and modern humans). It does not matter how long some survivors live after menopause, what is important is that most died before it.

If only one of these arguments is correct, the grandmother hypothesis has little value. The list can go on [45]. Some observations cannot be reconciled with the grandmother hypothesis. Older women have an increased chance of giving birth to twins and triples [46]. Furthermore, the outcome of such pregnancies in older mothers are better than in younger mothers [47, 48]. Why is declining fertility is associated with the increasing twinning rate? It is in agreement with hyperfunction theory. Hyperstimulation with FSH leads to multiple ovulation and a higher incidence of twins and triplets with age [46].

If menopause were adaptive, it would be conditional in the presence of grandchildren, but not in their absence. Conditional control is easy to achieve, even a single spike of sex steroids is sufficient to do the trick (this is exactly what a single pill of birth control pill like “*plan B*”, a progestin, does).

If nature equipped women with menopause to take care of grandchildren, why then does it impair their vision? Presbyopia, or age-related farsightedness, develops in humans by the age of female menopause. Is presbyopia an adaptive program as well? Like menopause, presbyopia is quasi-programmed; the ability to focus on near objects declines from childhood to adulthood, and its continuation culminates in presbyopia. By the time of menopause, presbyopia occurs in everyone. It is purely harmful and is treated by glasses (as a disease should be treated).

Male fertility gradually decreases with aging. Men do not have menopause, because men do not have a vulnerable menstrual cycle to start with (similarly, women do not have BPH).

Finally, consider a parody “grandfather hypothesis” that prostate hyperplasia develops in order to make men urinate in the middle of the night and thus protect grandchildren from lions. I hope it is not taken seriously, just as the bizarre grandmother hypothesis should not be either.

## Age-related diseases happen, potentially, in everyone

It is difficult to define a disease, especially an age-related disease [49, 50]. For example, osteoporosis and obesity were not officially recognized as diseases until 1994 and 2013, retrospectively. Whether we define age-related alterations as a disease depends on political, cultural, financial, medical and social reasons.

The main objection to considering age-related diseases such as menopause and presbyopia as diseases is that they happen to everyone. However, disease does not need to be rare to be a disease. For example, everyone may be sick with influenza during their lifetime, but it does not make it any less a disease. Furthermore, no definition of disease includes the requirement that it should not affect everyone.

All age-related diseases happen either in everyone (for example, prostate enlargement in men and atherosclerosis) or would happen in everyone (Alzheimer’s disease and cancer), if one does not die from a competing disease. For example, a human may suddenly die from myocardial fibrillation due to coronary atherosclerosis at the age of 60, but if one were saved and properly treated, they may be diagnosed with Alzheimer’s disease, and die from cancer at the age of 80.

## Age-related diseases are quasi-programmed

Age-related diseases occur to everyone, and, therefore, no one is immortal.

They happen in everyone because they are quasi-programmed in development, a continuation of growth and reproductive programs. External (environmental) factors and genetic predispositions also play a role, making certain age-related diseases manifest at different times or even not manifest at all in a lifetime.

For example, hypertension is a continuation of developmentally increased blood pressure from the newborn (blood pressure 64/41 mmHg) to the adult. Hypertension can also be viewed as a quasi-program of growth upon its completion. In fact, accelerated postnatal growth leads to higher blood pressure later in life [51]. Yet, external factors such as alcohol and smoking may accelerate the development of hypertension [52].

Cancers are the least quasi-programmed among all aging-related diseases because of the critical role of (a) external factors (e.g., smoking) that cause mutations and (b) inherited genetic susceptibility. In prostate enlargement, in comparison, environmental and genetic factors play a lesser role, and the prostate becomes hyperplastic and hypertrophic in everyone.

External factors and genetic variations may accelerate and aggravate quasi-programmed diseases. In humans, the role of external factors and genetic variability may obscure quasi-programmed nature of diseases. In genetically identical *C. elegans* at identical conditions, age-related diseases are clearly quasi-programmed [53–57].

## Age-related diseases are hyper-functional

Age-related diseases are driven by hyperfunctions on different levels: from signal-transduction pathways, to cells and tissues, to systems and organs. These hyperfunctions eventually damage tissues and organs, causing secondary loss of function. Hyperfunction is a function that was not turned off upon its completion [58]. (Note: Hyperfunction is not necessarily an absolute increase in function but may even be a decrease if it is still higher than optimal for longevity [59]). For example, mTOR drives cellular growth, but when the cell cycle is blocked, and mTOR is not turned down, then it drives the senescence phenotype associated with hyperfunctions such as SASP and proinflammation [60]. Cellular hyperfunctions are tissue-specific: osteoclasts resorb the bone, thus leading to osteoporosis; fibroblasts and immune cells cause proinflammation, associated with most age-related diseases; constriction of arterial SMC causes coronary artery spasm; blood platelets form clots. On systemic levels, hyperfunctions include hyperinsulinemia, hypertension, hyperglycemia, hyperlipidemia and others.

Cellular hyperfunction inevitably leads to age-related diseases and then to organ failure and secondary functional decline [61]. For example, hyperfunctional cells promote atherosclerosis, hypertension, arterial spasm, thrombosis, culminating in myocardial infarction, which, in turn, causes loss of function [20, 62].

mTORC1-dependent beta-cell hyperfunction culminates in beta-cell exhaustion (diabetes) [63–65]. Ovarian overactivation leads to follicular exhaustion and menopause [27, 30–32, 34, 36–38].

Hyperfunctional phases of pre-diseases are often asymptomatic, while their consequences – loss of function – are always symptomatic. Even classic diseases, such as hypertension, may have mild symptoms until damage occurs (stroke, myocardial infraction, heart or renal failure).

Functional decline in athletic performance [66] can precede official age-related diseases. Such an early-life decline is not caused by recognized age-related diseases. Early-life hyperfunctions are unrecognized. They are asymptomatic, until causing mild functional decline in athletic performance in everyone. Secondary loss of function can be observed early in life due to unnamed hyperfunctions.

## Is aging a disease?

According to conventional views, aging is a risk factor for developing disease. It is believed that aging can be healthy (without diseases) and that humans can die either from aging or from diseases. It was claimed, “aging should be strongly considered not to be a disease and as such should not be treated” [67].

According to hyperfunction theory, aging is not a risk factor, aging is the sum of all age-related diseases. There is no aging without these diseases. So-called “healthy” aging is slow aging observed in centenarians, who develop diseases later in life. But no centenarian dies from old age, all die from age-related diseases [68–70].

Like quasi-programmed diseases, aging is a natural continuation of developmental programs that were not switched off upon their completion. Aging is the sum of all quasi-programmed diseases. As David Gems put it, aging versus disease is a false dichotomy [71].

Aging is natural. Natural process is a disease, if it leads to death or functional decline [50, 71, 72]. A natural process, such as atherosclerosis, is a disease, whereas an unnatural process, such as a car accident, is not a disease. All age-related diseases are natural, and therefore we are mortal.

Aging is driven, in part, by hyperfunctional signaling pathways, such as the nutrient-sensing and growth-promoting mTOR pathway. Inhibition of the mTOR pathway by genetic, pharmacological and other means extends lifespan in numerous species and decelerates development of age-related diseases [73–75].

As suggested in 2006, “Once development is completed, a program for development is not switched off, thus becoming a quasi-program for aging. This hyper-functional quasi-program is manifested as diseases of aging, leading to organ damage and secondary decline.” [58]. (Note: Secondary decline is the most visible manifestations of advanced aging).

So, is aging a disease?

On one hand, aging is a progressing disease with 100% mortality rate. It can be treated (as a disease) with rapamycin, for instance. Diseases can be prevented by slowing down aging [58]. Potential anti-aging drugs could be tested by slowing diseases. Disease or not, aging is as treatable as a disease [76].

However, aging is not a specific disease, but the sum of all age-related diseases, including both life-limiting (e.g., diabetes, cancer and CVD) and non-life-limiting (e.g., osteoarthrosis and gray hair). It is a form of complex disease syndrome [71]. Using an analogy, is the American people a human? Is it a man or a woman? The people consist of all men and women; each of them is a human. But the people are not a human, neither a man nor a woman. Similarly, aging consists of all quasi-programmed alterations, age-related pre-diseases and diseases, early unrecognizable diseases that manifested as early functional decline, cosmetic conditions, and others. The aging process is the common mechanism of all diseases.

Given that aging is a sum of all age-related diseases, it can be called aging syndrome, or aging.

Aging seems mysterious, if one is studying so-called “healthy” or “successful” aging. One can subtract disease after disease until nothing is left. No aging. It is like subtracting every man and woman from the American people until nothing is left. Aging looks quasi-programmed, because it consists of quasi-programmed diseases that are driven by hyperfunctions that culminate into organ/system failure (and secondary loss of function). Aging behaves as the sum of all diseases. And this sum can be prevented by inhibiting the common mechanism that we call aging. Aging is driven by the same processes as diseases: from over stimulated signal-transduction pathways to cellular hyperfunction, systemic hyperfunction leading to organ failure (secondary functional decline). To understand aging, we should depict the pathogenesis of overlapping age-related diseases driven by hyperfunctional signals and cells towards organ damage. Aging is a collection of processes that drive quasi-programmed diseases. Preventive medicine that targets early hyperfunctional stages of a group of overlapping diseases is an anti-aging medicine. Aging can be understood through the development of all quasi-programmed diseases. Treatments that prevent age-related diseases partially prevent aging and *vice versa* [71, 77].
